# BSA Nanoparticles for siRNA Delivery: Coating Effects on Nanoparticle Properties, Plasma Protein Adsorption, and *In Vitro* siRNA Delivery

**DOI:** 10.1155/2012/584060

**Published:** 2012-08-07

**Authors:** Haran Yogasundaram, Markian Stephan Bahniuk, Harsh-Deep Singh, Hamidreza Montezari Aliabadi, Hasan Uludağ, Larry David Unsworth

**Affiliations:** ^1^Department of Chemical and Materials Engineering, Faculty of Engineering, University of Alberta, Edmonton, AB, Canada T6G 2G6; ^2^National Research Council, National Institute for Nanotechnology, Edmonton, AB, Canada T6G 2M9; ^3^Department of Biomedical Engineering, Faculty of Medicine and Dentistry, University of Alberta, Edmonton, AB, Canada T6G 2G6

## Abstract

Developing vehicles for the delivery of therapeutic molecules, like siRNA, is an area of active research. Nanoparticles composed of bovine serum albumin, stabilized *via* the adsorption of poly-L-lysine (PLL), have been shown to be potentially inert drug-delivery vehicles. With the primary goal of reducing nonspecific protein adsorption, the effect of using comb-type structures of poly(ethylene glycol) (1 kDa, PEG) units conjugated to PLL (4.2 and 24 kDa) on BSA-NP properties, apparent siRNA release rate, cell viability, and cell uptake were evaluated. PEGylated PLL coatings resulted in NPs with *ζ*-potentials close to neutral. Incubation with platelet-poor plasma showed the composition of the adsorbed proteome was similar for all systems. siRNA was effectively encapsulated and released in a sustained manner from all NPs. With 4.2 kDa PLL, cellular uptake was not affected by the presence of PEG, but PEG coating inhibited uptake with 24 kDa PLL NPs. Moreover, 24 kDa PLL systems were cytotoxic and this cytotoxicity was diminished upon PEG incorporation. The overall results identified a BSA-NP coating structure that provided effective siRNA encapsulation while reducing *ζ*-potential, protein adsorption, and cytotoxicity, necessary attributes for *in vivo* application of drug-delivery vehicles.

## 1. Introduction

Short interfering RNA (siRNA) is extremely promising for the therapeutic treatment of a myriad of diseases; however, its clinical application has hitherto been hindered by an apparent inability to control its delivery. The use of NP based drug delivery vehicles presents several advantages over conventional delivery stratagems, including the fact that they may be used for precise tissue targeting, remain in blood for a prolonged time, and be immediately injected into the systemic circulation. Furthermore, favorable tissue responses have been observed for decreasing particle sizes [1] and a multitude of covalent and noncovalent modifications of NP surfaces can be achieved, aspects that facilitate the design of more effective carriers. In particular, BSA-based NPs have many advantageous qualities [2]: presence of a hydrophobic core facilitating delivery of hydrophobic drugs, a natural abundance in plasma, relative stability and inertness in biochemical pathways, availability, and a relatively benign *in vivo* biological fate [3]. Unlike NPs fabricated from synthetic polymers, it is thought that the natural protein removal mechanisms will result in a reduced overall toxicity related to the application of BSA NPs [3]. That said, an important step in facilitating the localization of these NPs at the site of interest involves both decreasing their removal from the circulation (i.e., decreasing opsonization) as well as ensuring that any targeting moiety remains able to interact with the cellular site of interest. Inhibiting nonspecific protein adsorption will then be central to both of these effects. A common strategy for preventing protein adsorption at the tissue-material interface is to incorporate end-tethered PEG to the surfaces of biomaterials. It has been well established that the presence of end-tethered PEG can prevent particulate aggregation, reduce interactions with plasma proteins [4], minimize reticuloendothelial system clearance, and prolong blood circulation time of a host of NPs [5–7].

PEGylation of surfaces has been shown to impede nonspecific protein adsorption [8], where both the presence and conformation of end-tethered PEG play a critical role [9, 10]. It is noteworthy that not only is the amount of plasma protein adsorbed at the tissue-biomaterial interface important in obfuscating an engineered surface but also the composition of the protein layer itself is critical, as this may ultimately direct host responses. NP opsonization has been correlated to surface properties, including hydrophilicity, roughness, *ζ*-potential, and surface chemistry [11]. Recent results [12] have shown that systemic administration of BSA NPs, stabilized with polyethyleneimine-graft-PEG with bisphosphonic acid attached for bone targeting, showed no beneficial effects associated with the polymer coating. Although the reason for this result was not fully elucidated, it was postulated that the biodistribution of the NPs may be affected by the presence of the adsorbed protein corona to the PEG modified NPs; it is hoped that further analyzing the adsorbed protein composition to these PEG modified BSA NPs may clarify this point. Other previous work [13] has looked specifically at the use of positively charged poly-L-lysine(PLL) as a coating polymer that stabilizes the NP used for the apparent release of siRNA from BSA NPs. It was observed that, for low concentrations of PLL, varying the size of the PLL used for coating resulted in minimal effect on the net release of siRNA from the NPs. In further work [14], the release of a model drug from BSA NPs could be controlled from ~5 to 90% over 14 days, depending on the nature of coating designed to display differential stability against endogenous enzymes. 

In this study, we continued the development of BSA NPs by exploring the role of PEG coating by employing comblike structures of PEG-conjugated PLLs and compared systems stabilized *via* unmodified PLL. Specifically, PLLs of 4.2 and 24 kDa were utilized to understand the effect molecular weight may have on critical issues related to NP stabilization, siRNA encapsulation and passive release kinetics, plasma protein adsorption, cytotoxicity, and cellular incorporation. Conjugates of PLLs with 1 kDa PEG were synthesized so as to determine if any direct effect on NP stabilization as well as siRNA encapsulation and passive release kinetics might be altered. The cellular uptake of the NPs and the plasma protein adsorption profile were assessed, investigating the role of PEG coating on these features. Our results identified specific types of BSA NPs that provided adequate siRNA release and cellular uptake with relatively low amounts of protein adsorption and cytotoxicity. 

## 2. Materials and Methods

BSA and HBr salt of PLLs of different MWs (4.2 and 24 kDa) were purchased from Sigma-Aldrich (St. Louis, MO, USA) and used without further purification. The sodium dodecyl sulfate (SDS) was obtained from J. T. Baker (Phillipsburg, NJ, USA). FAM-labelled siRNA (double stranded, 21 base pairs) was purchased from Ambion Inc. (Austin, TX, USA). EDTA/trypsin (10X; Invitrogen, Carlsbad, CA, USA) was diluted 1 : 10 with Hank's Buffered Salt Solution (HBSS; Invitrogen) to 0.05 g/L concentration before use. Dulbecco's Modified Eagle Medium (DMEM; high glucose), and penicillin/streptomycin (10000 U/mL/10 mg/mL) solution were obtained from Invitrogen. Fetal bovine serum (FBS) was from PAA Laboratories (Etobicoke, Ontario, Canada). Sodium phosphate, monobasic, monohydrate sodium phosphate, and sodium chloride laboratory-grade reagents were purchased from EMD Chemical Inc. (Darmstadt, Germany). Ethanol was purchased from Fischer Scientific (Ottawa, Ontario, Canada). The N-hydroxysuccinimide ester of 1 kDa (mPEG-NHS) was obtained from Creative PEG works, NC, USA. The 3,3′,5,5′-tetramethylbenzidine substrate (TMBS) was obtained from Promega (Madison, WI, USA). The dialysis tubing of various MW cutoffs was obtained from Spectrum Laboratories Inc. (Rancho Dominguez, CA, USA).

### 2.1. PEG Conjugation to PLL

Previously reported methods were used to conjugate PLL to 1 kDa PEG [15]. Briefly, PLL was dissolved in 0.1 M phosphate buffer (PB, pH 7.4) to a final concentration of 2.4 mg/mL. mPEG-NHS was diluted to 18 mM using 0.1 M PB, added to the PLL solution, and reacted for 3 hrs at room temperature. Solution was dialyzed 2 days against MilliQ (18 Ω) water, at 4°C; MilliQ water being changed twice a day. Dialysate containing 4.2 and 24 kDa PLL was freeze-dried and used to reconstitute the polymer solutions at desired concentrations. 

### 2.2. NP Preparation

BSA NPs were formed *via* a coacervation method as detailed elsewhere [13]. Briefly, 250 *μ*L of 10 mg/mL BSA in water was added dropwise to an equal volume of 10 mM NaCl in water at room temperature. After 15 min stirring (600 rpm, room temperature), ethanol was added dropwise to a final volume ratio of 6 : 1, ethanol to BSA solution. The mixture was stirred for 3 hrs to form NPs. To stabilize formed NPs, native PLLs (0.3 mg/mL) or PEG-conjugated PLLs (0.3 mg PLL equivalents/mL) in deionized water were introduced dropwise to the equal volume of NPs suspension under constant stirring. Stirring was continued for 1 hr at room temperature to ensure time for the PLL and PEG-PLL conjugates to adsorb to the BSA NP surface. Suspensions of coated NPs were dialyzed against ddH_2_O for 3 days (12 hrs between solution replacements). Where indicated, the amount of coating on the surface was determined by using the FITC-labeled PLL or PEG-PLL. The polymer labeling was achieved by incubating 10 *μ*L of 0.1 mM FITC solution (in DMSO) with 1 mL of PLL or PEG-PLL (2 mg/mL in 100 mM phosphate buffer, pH = 7.4) for 1 hr at room temperature. Ethanol (9 mL) was then added to this solution. The solution then was centrifuged at room temperature (3000 rpm) for 15 minutes, and the supernatant, with unconjugated FITC, was removed. The pellet formed during this process was further washed with 5 mL of ethanol and centrifuged at 3000 rpm for 15 min [13]. The solids obtained were air-dried under vacuum for 5 hrs and stored in the dark at 4°C until used. The coating of FITC-labeled polymer was determined using fluorescence measurements. The coated NPs suspension was then diluted with phosphate buffer (pH = 7.4) by 100% and centrifuged (15,000 rpm, 1 hr) and fluorescence (*λ*_EX_: 485 nm; *λ*_EM_: 527 nm) of the supernatant was analyzed using a multiwall plate reader (Thermo Labsystems, Franklin, MA, USA). A calibration curve generated was used to calculate the coating efficiency as (1 − FITC-polymer_supernatant_/(FITC-polymer_supernatant_ + FITC-polymer_pellet_)) × 100%.

### 2.3. Particle Sizing and *ζ*-Potential

Mean particle size of the coated and uncoated NPs were determined using dynamic light scattering (Zetasizer 3000 HS, Malvern Instruments Ltd., UK) with a 633 nm He-Ne laser at a scattering angle of 90°. Uncoated BSA NPs were used directly for the measurements while the coated BSA NPs were diluted 1 : 2 with PB (10 mM, pH 7.4). Intensity measurements were used to determine the NP size. The *ζ*-potential of the NPs was determined by measuring their electrophoretic mobility using the same instrument at 25°C.

### 2.4. Plasma Incubation and Elution

Human blood plasma was obtained from Canadian Blood Services, where Canadian Blood Services obtained written informed consent from all volunteers for the collection and distribution of human blood products for research purposes. Human blood products were then shipped to our lab and used following research ethics procedures as approved by the University of Alberta Research Ethics Board (Institutional), Canadian Blood Services Research Ethics Board (Federal), and the National Research Council of Canada Research Ethics Board (Federal). NP solutions (500 *μ*L) were combined with an equal volume of undiluted human plasma and incubated at room temperature for 2 hrs with gentle rocking. The samples were then centrifuged at 13000 rpm for 10 minutes and the supernatants discarded. Pellets were resolubilized in 1 mL of 0.15 M phosphate buffered saline (PBS; pH 7.4) for 30 minutes and the procedure was repeated two times. These samples were spun down and pellets were solubilized in 2% w/v SDS in 0.15 M PBS for one hr, with rocking at room temperature in order to elute adsorbed plasma proteins. To separate the NPs from the eluted plasma proteins, the samples were centrifuged at 13000 rpm for 10 minutes and the supernatants collected and characterized using SDS-PAGE and immunoblotting analysis.

### 2.5. SDS-PAGE and Immunoblotting

Reduced SDS-PAGE and immunoblotting techniques were used to evaluate and to identify eluted proteins as described previously [9]. All electrophoretic apparatus were purchased from Bio-Rad (Hercules, CA). Briefly, samples were separated on 12% SDS-PAGE gels and then transferred onto a 0.2 *μ*m immunoblot PVDF membrane. The membrane was cut into strips for total protein staining using colloidal gold (Bio-Rad) and for immunoblotting. Primary and secondary antibodies (see Table S1 in Supplementary Materials available online at doi:10.1155/2012/584060) were used without further purification at concentrations of 1 : 1000. To visualize protein-antibody complexes, 350 *μ*L of stabilized TMBS substrate was incubated with membrane strips for 10 minutes at room temperature, with rocking. The colour-developing reaction was then quenched for 10 minutes using 2 mL of MilliQ water.

### 2.6. Preparation of siRNA Loaded BSA NPs and Release Studies

The coacervation technique previously described was employed to prepare BSA NPs encapsulating siRNA. Briefly, 500 *μ*L of aqueous solution of BSA (10 mg/mL) was added to an equal volume of 10 mM NaCl solution under stirring (600 rpm) in glass vials. The stirring was continued for 15 minutes at room temperature. To achieve siRNA encapsulation, siRNA solution (20 *μ*L of 0.15 mg/mL) was added to this binary solution and stirred at 600 rpm for 1 hr at room temperature. The siRNA (scrambled) used for encapsulation was either unlabeled or labeled with FAM. NPs were then formed by adding ethanol dropwise (final volume ratio of ethanol to starting BSA solution = 6). Stirring was continued for 3 hrs at room temperature after the complete addition of ethanol. To coat these siRNA loaded BSA NPs with PLL's, 500 *μ*L of an aqueous polymer solution was added dropwise to an equal volume of BSA NPs suspension under constant shaking of 500 rpm. Shaking was continued for 1 hr.

To obtain encapsulation efficiency, NPs containing FAM-labeled siRNA were centrifuged at 15000 rpm for 30 minutes. The FAM-labeled siRNA in the supernatant and pellet were determined using a plate reader (*λ*_EX_: 485 nm, *λ*_EM_: 527 nm) and a calibration curve based on known concentrations of FAM-labeled siRNA. The encapsulation efficiency was calculated as (FAM-siRNA_pellet_/(FAM-siRNA_pellet_ + FAM-siRNA_supernatant_)) × 100%. FAM-labeled siRNA was also used to study the apparent release kinetics of siRNA from BSA NPs coated with different polymers. Polymer concentration of 0.3 mg/mL was used to coat BSA NPs and release values were normalized to 0% for day 0, as described previously [13]. The suspensions were incubated at 37°C in PBS under shaking and aliquots were taken at predetermined time points and centrifuged at 15000 rpm for 30 minutes. The siRNA in the supernatant and pellet were determined using a calibration curve.

### 2.7. Cell Uptake Studies

To assess cellular uptake of NPs, human breast cancer MDA-231 cells were used. Two sets of NPs were prepared for cell uptake; (i) uncoated and coated BSA NPs with no siRNA, and (ii) uncoated and coated BSA NPs loaded with FAM-labeled siRNA. The siRNA encapsulation was achieved as described above, except that 20 *μ*L of siRNA solution (0.15 mg/mL) was used for FAM-labeled siRNA encapsulation. 1000 *μ*L of aqueous polymer solution was added to 2000 *μ*L of BSA NPs suspension (final concentration: 0.3 mg/mL) under shaking for 1 hr to achieve coating. The suspensions were then dialyzed against DMEM for 24 hrs with two changes in dialysis solution. 

For uptake, a monolayer of MDA-MB-231 cells were seeded in 24-well plates and allowed to attach for 24 hrs to reach ~50% confluency (see [16] for culture conditions on the cells). The medium was replaced with 500 *μ*L of fresh DMEM with 10% FBS and 1% antibiotics (penicillin/streptomycin). Then, 500 *μ*L of NP suspension in DMEM was added to the cells (in triplicate) and the cells were incubated for 24 hrs at 37°C in a humidified atmosphere of 95% air/5% CO_2_. After the incubation period, cells were washed with HBSS (×2) and trypsinized. A 3.7% formaldehyde solution was added to suspended cells and the siRNA uptake was quantified by a Beckman Coulter QUANTA SC flow cytometer using the FL1 channel to detect cell-associated fluorescence. The percentage of cells showing FAM-fluorescence and the mean fluorescence in total cell population were determined. Calibration was performed by gating with the negative control (i.e., “No Treatment”) group such that the autofluorescent cell population represented 1-2% of the total cell population.

## 3. Results and Discussion

The nature of the NP coating, in addition to its role in NP stabilization, is expected to control the apparent release of the encapsulated therapeutic agents whilst creating an interface that inhibits nonspecific protein adsorption. In addition to this, it is desirable to evaluate if PEGylation of the PLL based NP coating affects cytotoxicity and/or the cellular uptake of siRNA. Towards this end, NPs were coated with 4.2 and 24 kDa PLLs and PEG conjugates of the same. The use of PLL was considered advantageous as compared to previously employed PEI since the latter is synthetic, highly cytotoxic to mammalian cells, and undergoes an ill-defined degradation pattern. Unlike chemical crosslinkers, such as glutaraldehyde, the coating approach employed is thought to be a more bioacceptable means to stabilize the particles and further provides a convenient means of surface control. After characterization of the NP features, protein adsorption, composition of adsorbed protein layer, apparent siRNA release, cytotoxicity, and cellular uptake of siRNA wereassessed.

### 3.1. PEG-PLL Conjugation

Conjugation of mPEG-NHS to PLL was demonstrated through the addition of varying quantities of PEG and subsequent NMR analysis after dialysis to determine the resulting PEG:PLL molar ratio. The maximum conjugation ratio of PEG : PLL was determined to be ~26 and 150 for 4.2 and 24 kDa PLL systems, respectively (results not shown). On average, this translated into the incorporation of ~6 PEG per 1 kDa of PLL for both 4.2 and 24 kDa systems. However, given that the PDI of the PLL and PEG polymer was not one, these values are considered on average.

### 3.2. NP Characterization

The amount of PLL or PEG-PLL incorporated into the coating used to stabilize the NPs was determined so as to evaluate the effect of the presence of PEG upon *ζ*-potential and resulting NP size. It was evident that unmodified PLLs, regardless of size, incorporated similar mass amounts into the NP coating layer of ~0.1 mg PLL per mg BSA (Table 1). Although, it is evident that on a mole basis there would be more molecules of 4.2 kDa PLL adsorbed than 24 kDa systems, PLL adsorption is largely driven by electrostatic forces so the amount of PLL needed to occupy the charges on the surface of the NP was similar, regardless of individual chain lengths. Moreover, this amount of PLL in the coating layer agreed with previously published results for similar systems [13]. However, PEGylated 4.2 and 24 kDa PLL systems showed an adsorbed amount of PEG-PLL at 16 ± 6 and 7 ± 3 *μ*g conjugate per mg BSA, respectively. An order of magnitude decrease in adsorbed mass was seen in the stabilizing layer upon PLL PEGylation. It is likely that this significant decrease in adsorbed mass was due to steric hindrances imposed by preadsorbed PEG conjugates that prevented other PEG-PLLs from reaching the interface or from the screening of PLL charges that would reduce the driving force for PEG-PLL incorporation into the film. Differences observed between 4.2 and 24 kDa PEG-PLL coatings suggest that the smaller polymer conjugate may better fill the surface of the NP. 

 Mean particle sizes and *ζ*-potentials for PLL-coated NPs were similar to previously reported values (Table 1) [13]. NPs formed with 4.2 and 24 kDa PLLs yielded statistically similar average particle sizes of ~350 and 310 nm, respectively; results similar to previously reported values for PLL coated BSA NPs [13]. Upon using PEG-PLL for stabilizing the BSA NPs, the diameter of the NPs increased dramatically. For 4.2 kDa PEG-PLL systems a bimodal distribution in particle diameter was observed, where ~14 and 86% of the NP population had diameters of ~220 and 880 nm, respectively. Systems composed of 24 kDa PEG-PLL systems had an average diameter of ~845 nm. While the difference between systems using different PLL MWs was negligible, it is obvious that the differences observed upon incorporation of PEG were not. This large difference may be a direct result of the steric hindrances imposed by adsorbed PEG-PLL leading to lower amount of conjugate being incorporated into the stabilizing coating. With less conjugated PEG-PLL filling the surface, a larger NP may form.

 The *ζ*-potentials of all four types of NPs were positive, suggesting sufficient PLL or PEG-PLL adsorbed to offset the inherent negative *ζ*-potential of the BSA NP. The *ζ*-potential for the 4.2 and 24 kDa PLL systems were ~10 and 20 mV, respectively. The *ζ*-potentials for similar systems were found to plateau, with respect to increasing PLL concentration, around these values, suggesting that the NP surfaces were nearly saturated [13]. The PEG-PLL coating instead appeared to reduce the *ζ*-potential, as the 4.2 and 24 kDa PEG-PLL constructs had *ζ*-potentials of ~1.8 and 7.8 mV, respectively. Low average *ζ*-potentials for the PEG-PLL coated NPs seemed to suggest the presence of PEG, as PEG should result in a less charged surface as well as possibly screening *ζ*-potentials. It is interesting that there was a higher *ζ*-potential for the 24 versus 4.2 kDa PEG-PLL system given that the 4.2 kDa system adsorbed more PEG-PLL material. It may be that the more 4.2 kDa PEG-PLL molecules result in a more compressed PEG layer that shields the *ζ*-potentials of the PLL and thus lower the *ζ*-potential. Whereas the 24 kDa PEG-PLL film has more flexible PEG chains (i.e., mushroom regime) that may allow for more of the *ζ*-potential to be measured. The literature has shown that PEI-PEG systems observed a *ζ*-potential plateau at ~14 mV, which was greater than that observed herein [12]. A weak positive charge (*ζ*-potential < +5 mV) has been suggested for minimally adsorbing surfaces [17, 18] and PEG-coated NPs prepared in this study fulfill this feature, and surface PEGs could further improve the stability for such low *ζ*-potential NPs. Moreover, this low *ζ*-potential may also mediate NP aggregation; however, previous studies have shown that NP sizes are highly dependent on coating properties [13].

### 3.3. Protein Adsorption to NP Systems

The adsorption of proteins at the NP-blood interface is crucial to several important aspects of drug delivery, where a decrease in the amount of adsorbed protein may lead to an increase in the effectiveness of incorporating tethered targeting molecules on the NP surface. Moreover, decreasing protein adsorption may lead to increased circulation times by decreasing opsonization and potential host responses to the NP. Thus, in order to understand how the differences in the coating affects both nonspecific protein adsorption as well as the composition of the adsorbed layer, 4.2 and 24 kDa PLL and PEGylated versions of these PLLs were evaluated using platelet poor plasma adsorption, where adsorbed proteins were eluted from the surface using a 2% SDS incubation. As there is no way to accurately control the total surface area of NPs in solution, or to accurately estimate it, conducting a total protein analysis would not be indicative of the amount of adsorbed protein per surface area. Moreover, as it has been shown previously that 4.2 and 24 kDa PLL systems do not leak more than 1% of the BSA incorporated into the formed NPs within 2 hrs, all eluted proteins are most likely from adsorbed protein [13]. 

Since immunoblot analysis is qualitative for detecting protein levels, and more informative for determining protein presence, equal volumes of eluted protein solution were loaded (50 *μ*L), being a commonly employed technique. The results of the immunoblot analysis for the adsorbed plasma proteins on NPs are summarized in Table 2. The presence of high levels of fibrinogen and fibrinogen fragments suggests active coagulation in all samples except for the 24 kDa PLL NPs. It is possible that fibrinogen might have been less easily eluted from these surfaces. Adsorbed fibrinogen has previously been shown to activate platelets and induce the accumulation of phagocytes [19, 20].

 High intensity bands for human serum BSA were observed for all formulations. BSA adsorption in these systems is not unusual as the surfaces were formed with polymers that bind avidly to BSA. BSA is an unreactive protein that displays anticell adhesion and provides “passivation” properties, so that the presence of BSA in such great quantities on all of the NP systems is promising from a biocompatibility standpoint [21, 22].

Complement activation is a response against foreign surfaces with important implications for biocompatibility of administered agents [23]. Complement activation pathways are triggered by a variety of stimuli but ultimately serve to cause opsonization through the activation of C3 [24]. C3 is composed of *α* (115 kDa) and *β* (70 kDa) peptide chains. If complement is activated and the C3 cleaved, a 42-kDa fragment is created. The 42 kDa C3 fragment and the 70-kDa *β*-fragment were both present in relatively significant quantities on all NP systems. The presence of the 42-kDa C3 fragment indicated complement activation. PLL-coated NPs appeared to adsorb less 70 kDa C3. While this may be the case, it is important to consider that 70 kDa C3 may simply be less readily eluted from these surfaces than the others. The 115-kDa *α* C3 fragment was not observed on any of the systems. It is possible that the NPs did generate this fragment, but it did not adsorb to the NP surfaces.

Trace levels of apolipoprotein A-1 were found on both PLL-coated NP systems, while none was observed on PEG-PLL coated NPs. The presence of apolipoprotein A-1 implies anti-inflammatory activity. The literature states that HDLs such as apolipoprotein A-1 are also capable of endothelial protection, including the control of cell proliferation, the inhibition of apoptosis, the modulation of the secretory functions, the regulation of coagulation, fibrinolysis, and platelet adhesion, and the inhibition of inflammatory processes. Similar to the apolipoprotein A-1 case, 60 kDa plasminogen was found in moderate quantities on the 24 kDa PLL system, with minor amounts in 4.2 kDa PLL coated NPs and none on PEG-PLL coated NPs. This data suggests that PEG can effectively block plasminogen adsorption in these NP systems. Plasminogen binding is likely to be facilitated due to the epsilon amines of the PLL chains [25]. The presence of surface-localized plasminogen may actually enhance the biocompatibility characteristics of the NPs, as it is the precursor for plasmin, which has the potential for clot dissolution [25]. Further studies are needed to discern if the surface-adsorbed plasminogen indeed can be converted to plasmin within blood while localized to the NP surface. 

In addition to these five proteins, sixteen other proteins were screened without detection, even in trace amounts, for any of the NP formulations. These fifteen include high molecular weight kininogen (HMWK), low molecular weight kininogen (LMWK), factor I, fibronectin, *α*_1_-antitrypsin, thrombin, prothrombin, protein C, vitronectin, protein S, prekallikrein, antithrombin, immunoglobulin G (IgG), factor XII, factor XI, and *α*_2_-macroglobulin. The lack of the contact phase coagulation proteins of the intrinsic clotting cascade, prekallikrein, HMWK, factor XI and factor XII (Table 2) implied that the NPs should not be procoagulant. HMWK has been shown to both enhance biocompatibility through its anticell adhesion properties, as well as to hinder it by acting as a cofactor for the contact phase of coagulation; therefore, it is unclear whether the presence of this protein on the surfaces is desirable [26, 27]. Further along the cascade, the absence of prothrombin and thrombin reinforces the inference that the systems are noncoagulant [28]. Fibrinogen was detected in significant quantities (Table 2), so the absence of thrombin is especially important to prevent fibrin formation. 

The anticoagulation pathway was also monitored *via* the immunoblots. Protein C, a significant component of anticoagulation, was not observed in significant quantities. Protein S, a cofactor for Protein C, and vitronectin, an indirect inhibitor of plasminogen conversion to plasmin, were also not detected, indicating that the proteins controlling anticoagulation were not present. Two proteins involved in both coagulation and anticoagulation pathways, *α*_2_-macroglobulin and antithrombin, were investigated, but not detected again. Antithrombin is an uncharged serine protease inhibitor that is responsible for limiting irregular clotting [28]. Due to the absence of thrombin, the absence of *α*_2_-macroglobulin is inconsequential for the coagulation pathway. *α*_2_-Macroglobulin inhibits plasmin in the anticoagulation pathway, but was not detected. Antithrombin, which has a variety of targets in both the coagulation and anticoagulation pathways, was not observed in any of the NP systems.

The absence of other proteins not involved in clotting or fibrinolysis cascades is informative. Lack of IgG adsorption suggests a lack of reactivity by the circulating antibodies and no subsequent stimulation of the immune response. The lack of IgG also indicates that the possible complement activation seen (based on the presence of C3 fragments; Table 2) occurs *via* the alternative pathway only. The *α*_1_-antitrypsin is an important serine proteases in the body [29]. *α*_1_-Antitrypsin has a charge of −12 at a pH of 7.0, so its adsorption to the positively charged NPs would be expected. However, it was not detected in the immunoblot analysis. It is possible that other negatively charged proteins are preferentially adsorbed to the surface of the NPs, neutralizing its charge.

### 3.4. siRNA Encapsulation Efficiency and Release

**Figure 1:**
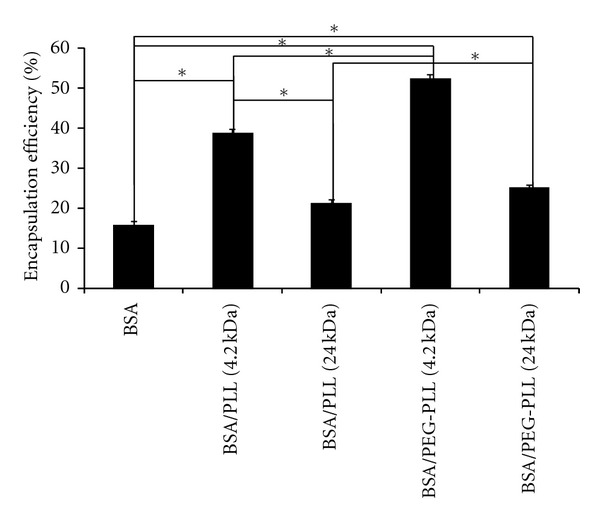
Encapsulation efficiencies of siRNA in various NPs. The value in parentheses represents the molecular weight of the PLL in kDa. For statistical comparison *via* double-sided *t*-tests, one asterisk (*) represents *P* < 0.05 and data represent average ±1 SD, *n* > 5.

siRNA encapsulation as a function of coating conditions was explored (Figure 1). Respective encapsulation efficiencies ranged from 16 ± 2% to 53 ± 7% for uncoated and 4.2 kDa PEG-PLL coated NPs. Statistically significant differences in encapsulation efficiency from uncoated NPs were observed for NPs with 4.2 kDa PLL (*P* < 0.005), 4.2 kDa PEG-PLL (*P* < 0.05), and 24 kDa PEG-PLL (*P* < 0.05). In almost all cases, the use of a coating increased the encapsulation efficiency. Previous studies have shown that when PEI was used to stabilize poly(D,L-lactide-co-glycolide) NPs, the encapsulation efficiency increased from ~43–80% [30]. The cationic polymers presumably sequester the siRNA from freely diffusing during the fabrication process and help to retain the therapeutic agent within the NPs. Using PEG-substituted PLL for coating resulted in increased encapsulation efficiency for both 4.2 and 24 kDa PLL systems. Furthermore, the 4.2 kDa PLL systems, with or without PEG, exhibited nearly double the encapsulation efficiencies of their corresponding 24 kDa PLL systems (Figure 1). Excluding the 24 kDa PLL systems (which did not give statistical significance from uncoated NPs), these results demonstrate that encapsulation efficiency can be controlled through varying PLL size and the incorporation of PEGylated PLL moieties.

**Figure 2:**
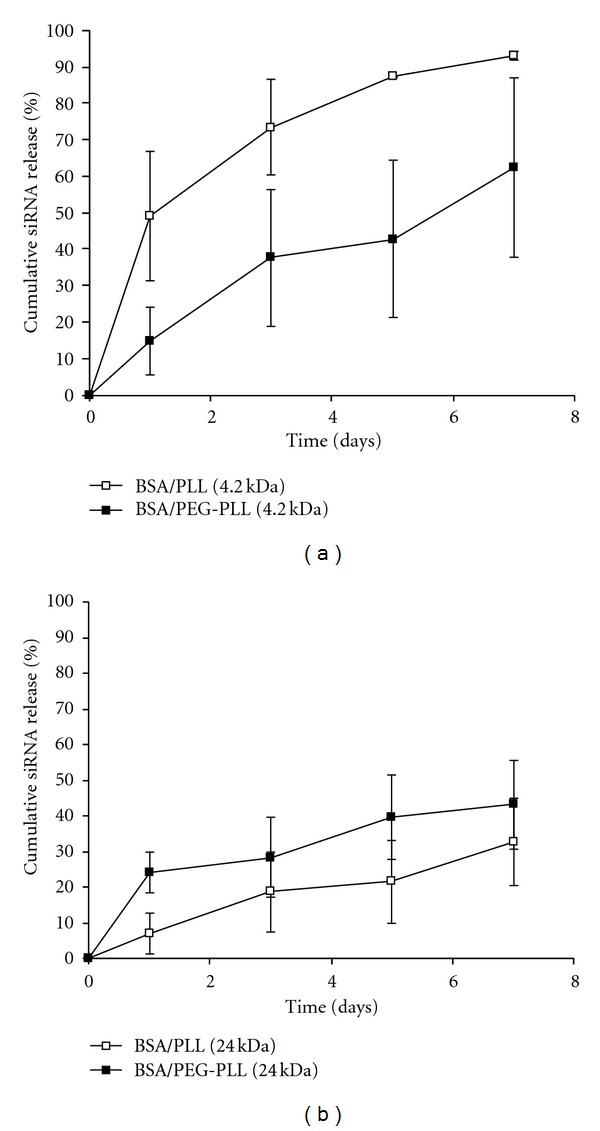
Cumulative siRNA release profile for 4.2 kDa (a) and 24 kDa (b) PLL-based coatings, over seven days. Trend lines are provided as a guide to the eye only. Data points represent an average ±1 SD, *n* ≥ 3.

The siRNA release from the NPs coated with PLL and PEG-PLL conjugates was monitored over 7 days (Figure 2). It should be noticed that all systems studied had a minimal burst effect, which may suggest the incorporation of the siRNA within the NPs studied. The highest release was observed in the 4.2 kDa PLL coated NPs, which had a Day 7 release of 93 ± 1%, while the lowest release was observed in the 24 kDa PLL coated NPs, with a Day 7 release of 33 ± 1%. Despite the similar adsorbed mass incorporated into the stabilizing layer for both 4.2 and 24 kDa PLLs, the release profile of siRNA was drastically different. This may be an indication that the 24 kDa PLL coatings form a more stable NP which may impede both the breakup of the NP and/or the diffusive release of siRNA. However, previous work in our lab has shown that 4.2 and 24 kDa PLL stabilized NPs yield similar stabilities at the 0.3 mg/mL condition [13]; thus, is it likely that the differences observed are most likely due to an increase in resistance to diffusive forces leading to a slower release profile for the 24 kDa PLL systems. 

Interestingly, the effect of incorporating PEG into the NP coating had a different effect upon siRNA release for 4.2 and 24 kDa PLL systems. After 7 days it was observed that 4.2 kDa coated NPs showed a decrease in siRNA release from 93 ± 1% to 62 ± 25% (*P* < 0.05) upon incorporating PEG, whereas for 24 kDa an increased release from 33 ± 1 to 43 ± 12% occurred upon PEG presence; the latter trend was not statistically significant. These data may coincide with the discussion regarding the density of the PEG-PLL layers highlighted by the *ζ*-potential studies. Namely, that the 4.2 kDa PLL layer adsorbed more PEG-PLL than the 24 kDa system, yet had a lower net charge that may suggest a denser PEG layer that shielded some of the 4.2 kDa PLL charge. Thus, it is probable that the large 24 kDa PEG-PLL conjugate was not as able to fill the surface an impede passive siRNA release as compared to the 4.2 kDa conjugate. Taken together, these results indicated that, based upon the presence/absence of PEG and the size of PLL, siRNA release from BSA NPs can be controlled over a range of ~20% to 90% over 7 days. Previously, mPEG-PGLA-PLL coated NPs [31] yielded a similar release profile, including ~85% release after 7 days. Related *in vivo* work using solid lipid NPs [32] had similar release profiles except for the fact that they observed an initial burst release of ~20% that was not observed herein. Through modifying NP creation parameters in this lipid study, the overall release could be varied from ~70–90% over a period of 7 days. An experiment involving PLGA-PLL NPs (with adsorbed PEG to improve circulation time) found a plateau in the release profile at ~55% after 7 days [33].

### 3.5. Cellular Uptake

**Figure 3:**
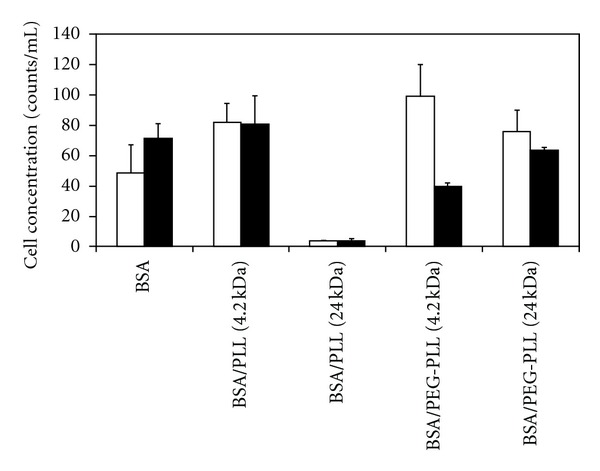
Cell concentrations after exposure to blank NPs (□) and FAM-siRNA-containing NPs (■). The value in parentheses represents the molecular weight of the PLL in kDa. The NPs coated in PEG-PLL (4 kDa) and containing FAM-siRNA showed the greatest cell concentrations. Data represent average ±1 SD, *n* > 5.

siRNA uptake was investigated in order to ascertain the delivery potential of the NPs as a function of coating properties. Flow cytometry was used to detect siRNA uptake based on NPs containing FAM-labeled siRNA, as well as the cell counts from the cultures exposed to the NPs. The latter is representative of cell survival upon incubation with the NPs containing no siRNA or FAM-labeled siRNA (Figure 3). Uncoated NPs and NPs coated with 4.2 kDa PLL had similar cell counts, irrespective of the presence or absence of encapsulated siRNA. Coating with 24 kDa PLL caused a dramatic drop in cell numbers, clearly indicating the toxicity of this type of coating irrespective of the presence or absence of encapsulated siRNA (Figure 3). After coating with PEG-substituted PLLs, there was little toxicity for the 24 kDa PLL for blank NPs and siRNA-containing NPs >20-fold increase in toxicity. With 4 kDa PLL, using PEG-substituted PLL gave better cells counts in the absence of siRNA but somehow reduced cell counts in the presence of siRNA (*P* < 0.05 between the two groups). No other system showed such a difference with and without siRNA (based on paired *t*-test). The encapsulated siRNA was nonspecific and was not expected on its own to cause cell toxicity. It is possible that it might have resulted in nonspecific effects once inside the cells since the molecule is highly charged and it might interact with cationic molecules critical for cell survival (such as histones, etc.), ultimately disrupting the normal cellular physiology. This issue needs to be further explored in future studies.

**Figure 4:**
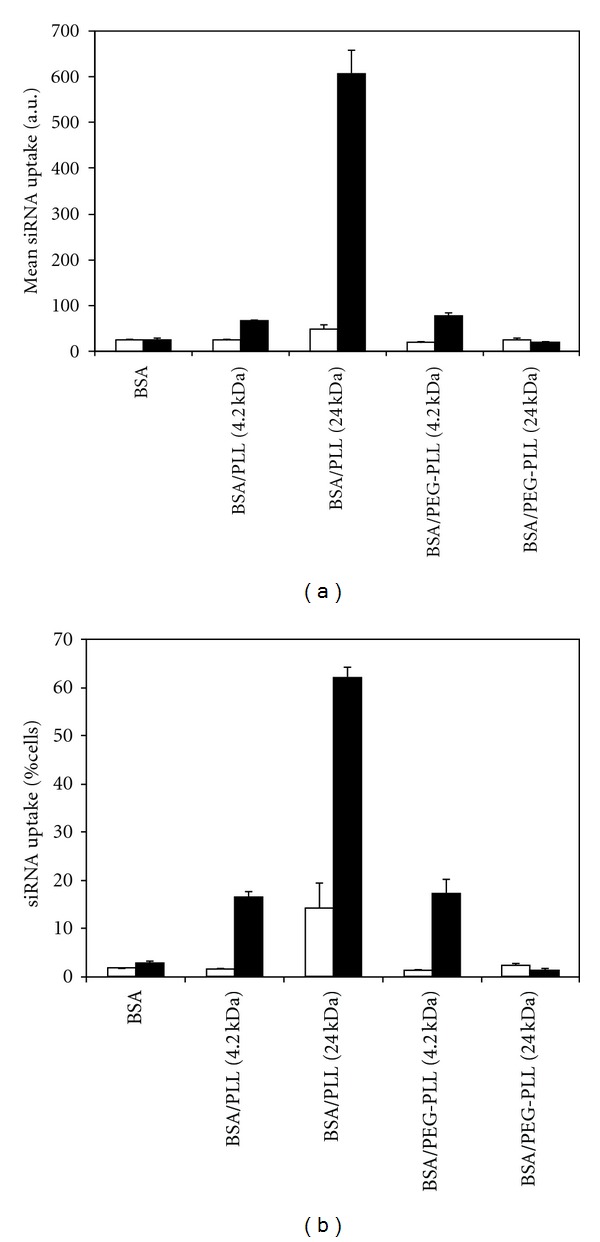
Data summarizing the mean uptake and percent cellular uptake of labeled and unlabeled siRNA. (a) Mean (+1 SD) FAM fluorescence of the cells exposed to NPs without siRNA (□) and with siRNA (■). BSA NPs coated with 24 kDa PLL showed the greatest cellular uptake. (b) Mean (+1 SD) siRNA-positive cells when the cells were exposed to NPs without siRNA (□) and with siRNA (■). BSA NPs coated with 24 kDa PLL showed the greatest value of siRNA-positive cell population. The value in parentheses represents the molecular weight of the PLL in kDa.

The siRNA uptake is summarized in Figure 4 as the mean uptake (Figure 4(a)) or the percentage of cells positive for siRNA (Figure 4(b)). The mean fluorescence of the cells exposed to NPs without FAM-siRNA was not statistically different among the NPs (as expected), and represented the background readings (i.e., normal autofluorescence). Compared to uncoated NPs, cells exposed to coated NPs containing FAM-siRNA all had greater fluorescence than the background (*P* < 0.01 for NPs coated with 4 kDa PLL, 24 kDa PLL and 4 kDa PEG-PLL), except the NPs coated with 24 kDa PEG-PLL. The latter did not show any evidence of increased uptake based on mean fluorescence of the cells. Although it is unknown why modification of the 24 kDa PLL NPs with PEG resulted in an insignificant amount of uptake (compared to controls), it is possible that the large average diameter of ~800 nm may prohibit cellular uptake. It was clear that the NPs coated with 24 kDa PLL had the highest cellular delivery of FAM-labeled siRNA. This was consistent with toxicity results that indicated highest toxicity (i.e., cell interaction) with this type of NPs. While the presence of PEG did not affect uptake with 4.2 kDa PLL, an apparent dramatic effect of PEG was evident with the 24 kDa PLL. The protein-repellent properties of PEG presumably prevented binding to cell surfaces, which is critical for internalization and siRNA uptake. This result was also in line with toxicity results, where the cells displayed much more tolerance to 24 kDa PEG-PLL coated NPs.

Figure 4(b) summarizes that uptake of BSA NPs among the cell population exposed to the NPs. Note that the uptake was minimal in the absence of coating (i.e., pure BSA NPs) and for NPs containing no FAM-labeled siRNA, which served as the background control (1–3% siRNA-positive cell population). The only exception to this observation was the NPs coated with the 24 kDa PLL; a high percentage of cells (14.4%) became auto-fluorescent that yielded significantly high proportion of “apparently” siRNA-positive cells. We previously made such an observation when NPs imparted certain toxicity on the cells [34]. For example, when cells are exposed to blank NPs with no reporter genes such as GFP, they display GFP-like fluorescence (with similar excitation/emission characteristics to FAM) even in the absence of a reporter gene. It is not surprising that the most toxic formulation in this study behaved in this way as well. 

With NPs coated by 4.2 kDa PLL and 4.2 kDa PEG-PLL, ~16 and 17% of the cells, respectively, yielded siRNA-positive cells, clearly indicating the beneficial effect of this coating on the cellular delivery of the NPs. With NPs coated by 24 kDa PLL, 62.2% of the cells yielded siRNA-positive cells, but using the same MW PLL with PEG substitution abolished the uptake totals (note the lack of difference in cell uptake for between siRNA-positive and siRNA-negative NPs). Considering the auto-fluorescence obtained in the cells exposed the 24 kDa PLL coated NPs, we expect the uptake to be closer to ~48% in this case. PEG obviously plays a significant role in this case, preventing the uptake of the NPs. This is in line with previous protein adsorption results, which indicated relatively less binding of plasma proteins to the NPs. It must be noted that the uptake values reported among the cell population should be considered as a relative measure to compare different NP formulations and not taken at absolute values. It is possible to significantly alter the measured values depending on the siRNA loading in NPs; with higher fluorescing NPs, higher rates of uptake could be obtained for the same formulations.

## 4. Conclusions

The stabilizing coating used on BSA NPs was expected to have significant implications on the physical characteristics of the formed NPs, blood plasma interactions, siRNA encapsulation, and cellular uptake. It was observed that the use of PEG increased average NP size and polymer coating on the NPs. In addition, PEG coatings were found to decrease nonspecific protein adsorption from human plasma as well as decrease the cytotoxicity of certain NPs (i.e., ones coated with highly toxic 24 kDa PLL). This result was likely not due to size, but rather attributed to inherently higher toxicity of the high MW PLL. Although an extensive array of adsorbed proteins was found on the NP surfaces, proteins for both passivating the NPs as well as activating the foreign body reactions were noted. The benefit of a PEGylated NPs was not clear in this respect and further evaluation (*in vitro* or *in vivo*) will be necessary to fully reveal the biocompatibility of the NPs. For the NPs coated with 4.2 kDa PLL, PEG also increased the siRNA encapsulation efficiency, maintained a similar cellular uptake, and delayed siRNA release over a period of 7 days. These desirable results suggest that NP engineering could be possible by controlling PLL size and the use of PEG to prevent the removal from the bloodstream without hindering the efficiency of drug delivery. It is thought that the fundamental knowledge acquired in this study will further the design of coating strategies for controlling the formation and biological interactions of BSA NPs in circulation for the express purpose of delivering drugs *via* systemic administration.

## Supplementary Material

A list of primary and secondary antibodies used for Western blot (immunoblot) analysis. Molecular weight, isoelectric point, and source are tabulated.

## Figures and Tables

**Table 1:** Selected characteristics of prepared NPs: (i) efficiency of PLL and PEG-PLL coating on BSA NPs, (ii) *ζ*-potential, and (iii) average size (average ± standard deviation, *n* ≥ 5). Note that the NPs from 4.2 kDa PLL-PEG coating gave two size populations with relative ratios of 14% and 86%.

	Coated amount (mg conjugate/mg BSA)	*ζ*-Potential (mV)	Diameter (nm)
Uncoated	—	−10.1 ± 0.4	255 ± 30
4.2 kDa PLL	0.12 ± 0.03	10.1 ± 0.5	350 ± 90
24 kDa PLL	0.10 ± 0.02	20.4 ± 0.5	310 ± 30
4.2 kDa PEG-PLL	0.016 ± 0.006	1.8 ± 0.3	220 ± 26 (14%)
			880 ± 100 (86%)
24 kDa PEG-PLL	0.007 ± 0.003	7.8 ± 0.9	845 ± 140

**Table 2:** BSA NP plasma adsorption conditions and results as evaluated with immunoblots.

	Fragment size (kDa)	Fragment name	System			
			4.2 PLL	24 PLL	4.2 PEG-PLL	24 PEG-PLL
Fibrinogen	48	*γ*	^ ∗∗∗^	^ ∗∗^	^ ∗∗^	^ ∗∗∗^
	56	*β*	^ ∗∗∗^	^ ∗∗^	^ ∗∗∗^	^ ∗∗∗^
	68	*α*	^ ∗∗∗^	^ ∗∗∗^	^ ∗∗∗^	^ ∗∗∗^
	<48	Cleavage	^ ∗^	0	^ ∗^	^ ∗∗^
Albumin	66		^ ∗∗∗^	^ ∗∗∗^	^ ∗∗∗^	^ ∗∗∗^
C3	42	Activation	^ ∗∗^	^ ∗^	^ ∗∗^	^ ∗∗^
	70	*β*	^ ∗^	^ ∗∗^	^ ∗∗^	^ ∗∗^
	115	*α*	0	0	0	0
Apolipoprotein A-1	27		0+	0+	0	0
Plasminogen	25		0	0	0	0
	60		0+	∗	0	0

Plasma proteins eluted with 1.0 mL of 2% SDS in PBS; 0 indicates zero band intensity, while 0+ indicates trace band intensity and ^∗∗∗^indicates highest intensity bands. Proteins shown in Table 1 but absent from this table were not observed in immunoblotting (zero band intensity throughout).

## Abbreviations

BSA: Bovine serum BSA
FAM: 5-Carboxyfluorescein
MW: Molecular weight
NP: Nanoparticle
PEG: Poly(ethylene glycol)
PLL: Poly-L-lysine
siRNA: Short interfering RNA.
