# Supplementary material for: BSA Nanoparticles for siRNA Delivery: Coating Effects on Nanoparticle Properties, Plasma Protein Adsorption, and In Vitro siRNA Delivery
Source: Int J Biomater. 2012 Aug 7;2012:584060. doi: 10.1155/2012/584060 (PMC3420107; doi:10.1155/2012/584060)
Supplement: Supplementary file 1 — A list of primary and secondary antibodies used for Western blot (immunoblot) analysis. Molecular weight, isoelectric point, and source are tabulated. [file 584060.f1.docx]

**Table 1**. Primary antibodies^a^ used for immunoblotting of plasma proteins adsorbed to coated NPs, along with the protein molecular weight, protein isoelectric point, host animal used for antibody generation, and the source of the antibody.

| Antibody | Protein MW (kDa) | Protein  pI | Host | Source |
| --- | --- | --- | --- | --- |
| Kininogen (LMWK) | 50 | 6.6 | Mouse | US Biological, Swampscott, MA, USA |
| Kininogen (HMWK) | 88-120 | 6.8 | Mouse | US Biological, Swampscott, MA, USA |
| Factor I | 88 | 7.6 | Mouse | Cedarlane Laboratories, Hornby, ON, Canada |
| Fibrinogen | 340 | 6.6 | Rabbit | Calbiochem, Gibbstown, NJ, USA |
| Fibronectin | 440 | 5.7 | Rabbit | Cedarlane Laboratories, Hornby, ON, Canada |
| α_1_-Antitrypsin | 47 | 5.6 | Sheep | Cedarlane Laboratories, Hornby, ON, Canada |
| Thrombin | 36 | 5.4 | Sheep | Cedarlane Laboratories, Hornby, ON, Canada |
| Prothrombin | 72 | 5.9 | Sheep | Cedarlane Laboratories, Hornby, ON, Canada |
| Protein C | 62 | 6.3 | Sheep | Cedarlane Laboratories, Hornby, ON, Canada |
| Vitronectin | 75 | 5.8 | Sheep | Cedarlane Laboratories, Hornby, ON, Canada |
| Protein S | 69 | 5.7 | Sheep | Cedarlane Laboratories, Hornby, ON, Canada |
| Prekallikrein | 85 | 8.2 | Sheep | Cedarlane Laboratories, Hornby, ON, Canada |
| Antithrombin | 53 | 6.7 | Sheep | Cedarlane Laboratories, Hornby, ON, Canada |
| IgG | 174 | 8.4 | Goat | Sigma-Aldrich, St. Louis, MO, USA |
| Plasminogen | 90 | 7.3 | Goat | Cedarlane Laboratories, Hornby, ON, Canada |
| Complement Factor 3 | 185 | 6.4 | Goat | Calbiochem, Gibbstown, NJ, USA |
| Factor XII | 80 | 7.7 | Goat | Cedarlane Laboratories, Hornby, ON, Canada |
| Factor XI | 160 | 8.1 | Goat | Cedarlane Laboratories, Hornby, ON, Canada |
| Apolipoprotein A1 | 28 | 7.1 | Goat | Sigma-Aldrich, St. Louis, MO, USA |
| α_2_-Macroglobulin | 718 | 6.4 | Goat | Sigma-Aldrich, St. Louis, MO, USA |

^a^ Horseradish Peroxidase Conjugate
